# Beta1 subunits enable fentanyl‐induced BK channel activation and opioid regulation of cerebral artery diameter

**DOI:** 10.1002/2211-5463.70347

**Published:** 2026-10-24

**Authors:** Shiwani Thapa, Mohammed Nooh, Akhil Geddati, Alex Dopico, Anna N. Bukiya

**Affiliations:** ^1^ Department of Pharmacology, Addiction Science and Toxicology, College of Medicine The University of Tennessee Health Science Center Memphis USA; ^2^ Department of Biochemistry Faculty of Pharmacy Cairo University Egypt

**Keywords:** cerebrovascular, MaxiK, opioid crisis, overdose

## Abstract

Ultra‐potent synthetic opioids are powerful analgesics but also a leading cause of drug overdose deaths. Calcium−/voltage‐gated potassium channels of large conductance (BK) control vital physiological processes, with their accessory β1 (encoded by *Kcnmb1* in mice) subunit being predominantly expressed in smooth muscle. Thus, we hypothesize that BK β1 determines the effect of fentanyl on cerebral artery diameter. Here we report that fentanyl (0.001–10 μm) activates BK heteromers consisting of slo1 + β1 subunits in CHO cells, but not homomers of slo1. In *ex vivo* pressurized mouse cerebral arteries, pharmacological inhibition of BK or β1 genetic deletion (*Kcnmb1* ^*−/−*^) led to fentanyl‐induced vasoconstriction. Naloxone, an opioid receptor antagonist, ablates the fentanyl response in wild‐type cerebral arteries whereas mild constriction persists in *Kcnmb* *1* ^*−/−*^ arteries. Therefore, we identify BK β1 as a functional fentanyl target which acts independently of opioid receptors to regulate fentanyl action on cerebral artery diameter.

## Graphical abstract

During the current opioid crisis, the majority of overdose deaths are attributed to ultra‐potent synthetic opioids, such as fentanyl. While fentanyl exerts its action via opioid receptors, we propose that potassium channels in the brain blood vessels may also sense fentanyl. We identified a specific protein—beta1 subunit—within the potassium channel protein complex as a new target of fentanyl that controls the diameter of brain arteries.

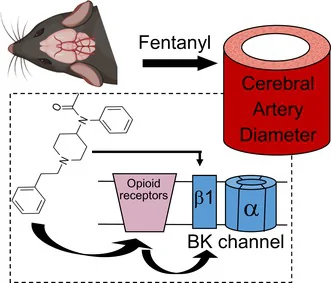

**Abbreviations**
BK: calcium‐ and voltage‐gated potassium channels of large conductance
CHO: Chinese hamster ovary
EC_50_
: effective concentration required to reach half‐maximal activation of BK currents
EC_max_
: effective concentration at which effect reaches its maximum
MCA: middle cerebral artery
UPSO: ultra‐potent synthetic opioids

Medical dispensing of opioids remains high, with approximately 125 million opioid prescriptions being fulfilled each year [1]. Within the ultra‐potent synthetic opioid (UPSO) family, fentanyl is approved for use during major surgeries (orthopedic procedures, open‐heart surgeries, etc.) to counteract the stress effect [2, 3, 4, 5, 6, 7]. Worldwide, UPSOs are utilized as stress‐relieving and analgesic intervention during fetal procedures *in utero* [8, 9], early stages of labor [10], and in controlling intense pain [11]. As opposed to chronic opioid (mis)use, the effects of episodic opioid exposure are much less studied. This type of exposure may occur not only during medical procedures but also as part of a national security threat event, intended poisoning, or unintended contamination of other drugs [12]. The concern with UPSO use rises from the narrow safety margin of these drugs, with fentanyl being the leading cause of overdose deaths [13]. Given the medical demand and the potential for intentional or accidental exposure to fentanyl, identification of the molecular targets driving this UPSO's end‐organ effects is critically needed.

Opioids generally trigger a drop in systemic arterial pressure leading to autoregulatory cerebral vasodilation to maintain blood flow to the brain [14]. However, several reports document that fentanyl constricts cerebral arteries. Blinded infusion of fentanyl in humans without brain abnormalities resulted in a 25% increased blood velocity of middle cerebral arteries (MCA), as measured by Doppler ultrasonography [15]. Increased blood velocity in a large artery usually reflects vasoconstriction. A consistent increase in mean cerebral blood flow velocity in MCA was also observed in awake humans following fentanyl administration [16, 17]. However, another study reported lack of fentanyl effect on MCA blood flow velocity [18]. Literature review concludes that “the cerebral effects of opioids are dependent on several factors, e.g., age, species, ventilation, anesthesia before and during measurements, systemic hemodynamics, and underlying diseases” [19]. Moreover, fentanyl effect on cerebral blood flow is reported to be region‐specific [20]. Variable effects of fentanyl on cerebral circulation that are seemingly disconnected from modifications in systemic blood pressure suggest involvement of multiple, local mechanisms. Although three opioid receptor types display high affinity for UPSO, recent evidence emerges that opioids may target ion channels directly, that is, independently of drug‐opioid receptor interactions, resulting in functional consequences [21].

MCA provides blood supply to the largest cortical territory of the brain compared to other cerebral arteries stemming from the circle of Willis [22]. While MCA diameter and smooth muscle tone are regulated by endothelial, neural, and circulating factors, they are primarily determined by ion channel activity in the smooth muscle membrane itself. Among cerebral artery smooth muscle ion channels, calcium‐ and voltage‐gated potassium channels of large conductance (BK) play a key role [23]. Upon activation, BK generates K^+^‐currents that repolarize the smooth muscle plasma membrane and ultimately lead to smooth muscle relaxation [24]. In cerebral artery smooth muscle, BKs are hetero‐oligomers that include channel‐forming α (“slo1”) and accessory β1 subunits [25]. The prevalent slo1 isoform in rat cerebral artery smooth muscle, cloned from freshly isolated smooth muscle cells, is termed “cbv1” (from “cerebrovascular”) and is > 95% conserved with mouse and human isoforms [26]. β1 subunits do not form channels themselves but largely determine the BK phenotype [23, 25]. β1 subunit, but not other betas, is abundant in smooth muscle (particularly in cerebral vasculature) and scarce in other tissues [25]. Several endogenously produced and exogenous compounds (such as estradiol, HENA, progesterone, alcohol, and lithocholic acid) were shown to modulate BK channel activity and artery diameter via BK β1 subunits [27, 28, 29, 30, 31]. The role of BK β1 subunit in sensing and mediating cerebrovascular actions of fentanyl remains unexplored.

In this study, we aimed at determining the role of BK β1 subunits in fentanyl effect on cerebral artery diameter. High‐throughput patch‐clamp demonstrated that fentanyl directly activated recombinant BK heteromers consisting of cbv1 + β1 subunits but not homomeric cbv1 channels. *Ex vivo* diameter tracing of pressurized MCAs from male and female C57BL/6J mice revealed sexually dimorphic cerebrovascular responses to fentanyl, with vasodilation being observed in males. Interestingly, genetic deletion of *Kcnmb1* or pharmacological inhibition of BK channels with paxilline modified fentanyl effect on cerebral artery diameter in both sexes. Following confirmation of opioid receptors' transcript presence in mouse MCAs, we demonstrate that the opioid receptor antagonist naloxone modifies fentanyl effect on artery diameter, yet the difference between wild‐type and *Kcnmb1* ^*−/−*^ MCA responses to fentanyl persists. Together, these findings identify BK β1 as an important determinant of the cerebrovascular response to fentanyl.

## Material and methods

### Recording of BK channel activity via robotic patch‐clamp

The Chinese hamster ovary (CHO) cell line was purchased from the European Collection of Authenticated Cell Cultures (ECACC, cell line CHO‐K1, catalog No: 85051005). Authenticity of cells was confirmed by the lack of measurable BK currents in non‐transfected cells and by BK currents characteristics in transfected cells when compared to other groups reporting BK channel expression in this cell line. All experiments were performed in mycoplasma‐free cells. These quality controls were carried out within the past three years. CHO cells were stably transfected with pcDNA3.1(+) that contained an insert coding for cbv1 (AY330293 [26]) or were co‐transfected with the pcDNA3.1/Hygro(+) vector carrying an insert for BK β1 subunit (FJ154955 [28]). Stable cell lines were grown in Ham's F‐12 cell media (Sigma‐Aldrich) supplemented with 10% (v/v) heat‐inactivated fetal bovine serum (FBS; GeminiBio), 1% (v/v) penicillin–streptomycin (Sigma‐Aldrich), and 1 mg/mL G418 ± 300 ug·mL^−1^ hygromycin B. Transfections were performed using X‐tremeGENE HP reagent (Roche). Recordings were performed in the whole‐cell patch configuration using Patchliner (Nanion) with the stability of the giga‐seal monitored throughout the recording as described [32]. Intracellular solution contained (mm) 25 KF, 30 K_gluconate_, 60 KCl, 5 NaCl, 15 HEPES, 7.5 HEDTA, 8.5 CaCl_2_, while extracellular solution contained (mm) 140 NaCl, 4 KCl, 1 MgCl_2_, 2 CaCl_2_, 10 HEPES. Ca^2+^_free_ in internal solution was 1–5 μm as validated by Ca^2+^‐selective electrode (Thermo Fisher Scientific). Currents were held at −80 mV, then stepped to +80 mV to record fentanyl effect before returning to holding level. Fentanyl was applied extracellularly.

### Pressurized cerebral artery diameter measurements

Eight–12 weeks‐old C57BL/6J mice and *Kcnmb1* ^*−/−*^ on C57BL/6J background were bred from the same parental pair (male and female), and each *Kcnmb1* ^*−/−*^ versus wild‐type offspring was confirmed by genotyping. All animal activities were reviewed and approved by the University of Tennessee Health Science Center Institutional Animal Care and Use Committee (protocol #25–0607). University of Tennessee Heath Science Center is an Association for Assessment and Accreditation of Laboratory Animal (AAALAC)‐accredited institution. Animals were housed in ventilated cages on a 12‐h light cycle (lights off at 6 pm). They received water and standard rodent chow *ad libitum*. All husbandry and experimental procedures were performed by trained personnel to reduce animal suffering and anxiety. Vaginal cytology was used to determine estrous cycle, and females identified as being in estrus were excluded from experiments to avoid estrogen modulation of BK channel activity [33, 34, 35]. Vaginal swabbing for cytology evaluation was performed when female mice were anesthetized with inhalational isoflurane. For cerebral artery harvesting, mice were deeply anesthetized by brief inhalational exposure (~30 s) to isoflurane in an induction jar and subsequently euthanized by decapitation using sharp scissors. Middle cerebral arteries (MCAs) were dissected out and cannulated in a perfusion chamber bathed with physiological saline solution of the following composition (mm): 119 NaCl, 4.7 KCl, 1.2 KH_2_PO_4_, 1.2 MgSO_4_, 0.023 EDTA, 11 glucose, 24 NaHCO_3_, 1.6 CaCl_2_. Saline was equilibrated at pH 7.4 with a 21/5/74% mixture of O_2_/CO_2_/N_2_ gases and maintained at 35–37 °C. After mounting, arteries were maintained at 0 mmHg for approximately 10 min, pressurized to 10 mmHg for 10 min, and then pressurized to 60 mmHg for 35 min to trigger development of steady myogenic tone. Thus, all arteries underwent a standardized equilibration and stepwise pressurization period of approximately 55 min before drug application. Arteries were randomized, yet it was ensured that arteries from the same donor were not included into the same group. Artery diameter was continuously monitored via camera mounted on a microscope, and external diameter value was digitized using IonWizard software (IonOptics). Drugs were dissolved in physiological saline solution and perfused via chamber. The effect of drug on arterial segments was evaluated at the time the external diameter reached a maximal, steady level immediately prior to washout.

### qPCR

Total RNA extraction from mouse tissue samples was conducted using the RNeasy Plus Micro Kit (Qiagen). RNA concentration was determined by a NanoDrop Spectrophotometer (ThermoFisher Scientific). For cDNA synthesis, the High‐Capacity cDNA Reverse Transcription Kit (Applied Biosystems) was used in a ProFlex PCR system (Applied Biosystems). qPCR was conducted on a QuantStudio 5 system (ThermoFisher Scientific) using TaqMan Fast Advanced Master Mix and TaqMan gene expression assays (ThermoFisher Scientific). All samples were run in triplicates for both target and reference (*Gapdh*) genes, following manufacturer protocols. Gene expression levels were quantified using the 2^−ΔCt^ method [36, 37]. Results from each triplicate were averaged to represent a single data‐point.

### Chemicals

All chemicals were purchased from Sigma‐Aldrich. Blood gas mixture (O_2_/CO_2_/N_2_) was purchased from Nexair (Memphis, TN). Fentanyl was first prepared as a 600 μm stock solution in either robotic patch‐clamp external solution or in physiological saline solution for pressurized arteries. Then, stocks were diluted in respective solutions to render final concentrations. Naloxone was prepared as a 1 mm stock solution and then diluted to render a 10 μm final working concentration.

### Data analysis

Final plotting and fitting of data were conducted using Origin 2020 (OriginLab). All data were expressed as means ± standard error. Concentration‐response data‐points from patch‐clamp were fitted by a biHill function: y = 1/(1 + (0.015/x)^1.7^)/(1 + (x/10)^1.0^). Concentration‐response curves from artery diameter measurements were fitted by a BoltzV function of the following type: y = ((A_1_−A_2_)/(1+ e^(x + x0)^)) + A_2_.

Statistical analysis was carried out using free online resource statskingdom (https://www.statskingdom.com/). Comparison of 2 groups of data was performed using two‐tailed Welch's *t*‐test. For analysis with 2 independent variables, two‐way analysis of variance (ANOVA) with Tukey *post hoc* test was used. *P* < 0.05 was considered statistically significant.

## Results

### Fentanyl activates BK currents via β1 subunits

Using high‐throughput robotic patch‐clamp (Patchliner by Nanion), we studied the action of fentanyl on BK channels stably expressed in CHO cells. Channels consisted of either slo1 subunits (cerebrovascular isoform cloned from rats, cbv1 [26]) or cbv1 co‐expressed with BK β1 subunit cloned from the same species [28]. CHO cells have undetectable‐to‐low levels of opioid receptor transcripts (Fig. 1A). This is consistent with previous reports that CHO cells largely lack opioid receptors [38]. CHO cells also lack endogenous BK channels and are widely used for studying BK channel pharmacology [39, 40]. Fentanyl was applied extracellularly to whole‐cell patch configuration at a range that included pharmacologically relevant, progressively increasing concentrations: 0.0001, 0.001, 0.01, 0.1, 1, and 10 μm. In whole‐cell patch configuration, G‐protein signaling is disabled, as the cytosolic milieu is diluted into pipette solution (see composition in Methods). Free calcium in the intracellular (pipette) solution was set at low μm level (≈3 μm) as micromolar calcium concentration is estimated to be reached in the vicinity of BK channel during cerebrovascular myocyte contraction [41]. As evident from Fig. 1B‐D, fentanyl activates β1 subunit‐containing channels (cbv1 + β1) but not β1‐lacking cbv1. In cbv1 + β1 channels, fold‐change increase in BK current amplitude in presence of 0.1 μm fentanyl averages 11.22 ± 3.47 from pre‐fentanyl levels whereas in homomeric cbv1 channels fold‐change in BK current averages 1.54 ± 0.29 (*P* < 0.05 by unpaired *t*‐test, Fig. 1C). Concentration‐response curves for fentanyl on cbv1 + β1 channels could be best fitted with biHill function (Fig. 1D). From this fit, effective concentration required to reach half‐maximal activation of BK currents (EC_50_) by fentanyl is 0.015 μm. Maximal increase in BK current amplitude is observed at 0.2 μm fentanyl. Altogether, our data support the idea that BK β1 is the functional target that enables fentanyl activation of BK channels.

**Fig. 1:**
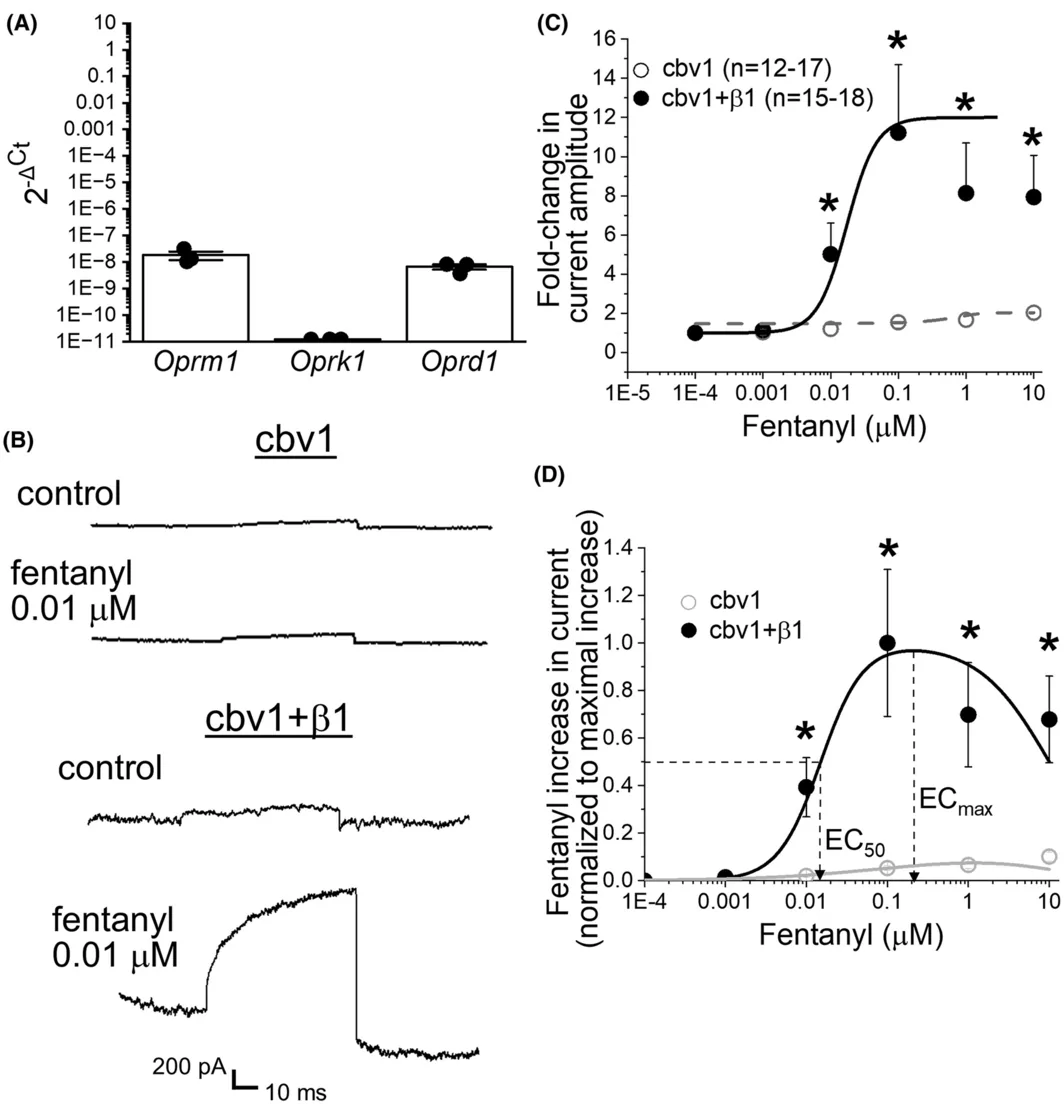
β1 subunit confers fentanyl sensitivity to calcium‐ and voltage‐gated potassium channels of large conductance (BK). (A) Opioid receptors mu, kappa, and delta (*Oprm1, Oprk1*, and *Oprd1*) transcripts are lacking in lysate from non‐transfected Chinese Hamster Ovary (CHO) cells. TaqMan probes used included: Mm01188089_m1 (*Oprm1*), Mm01230885_m1 (*Oprk1*), and Mm01180757_m1 (*Oprd1*). Glyceraldehyde‐3‐phosphate dehydrogenase gene transcript *Gapdh* (probe Mm99999915_g1) was used as a reference gene. Here and in C, D, error bars indicate standard error of the mean (SEM). (B) Original records showing fentanyl action on currents generated by β1‐lacking (cbv1, top) versus β1‐containing (cbv1 + β1, bottom) calcium−/voltage‐gated potassium channels of large conductance (BK) expressed in CHO cells. Currents were obtained in whole‐cell configuration, V_hold_ = −80, V_step_ = +80 mV, Ca^2+^_free_ = 3 μm. Fentanyl was applied from the outside of the cells. (C) Dose–response curve. Here and in C, *P < 0.05 compared to effect on β1‐lacking channels (unpaired *t*‐test). (D) Dose–response curves normalized to maximal effect of fentanyl across both channel constructs. For β1‐containing channels, data were fitted using a biHiIl function. Effective concentration needed to reach half‐maximal effect (EC_50_) ≈ 0.015 μm, effective concentration needed to reach maximal effect (EC_max_) = 0.2 μm.

### BK β1 subunit participates in fentanyl effect on cerebral artery diameter

To determine whether BK β1 subunit contributes to fentanyl effect on cerebral artery diameter, we probed this opioid on *ex vivo* pressurized MCA harvested from C57BL/6J mice (wild‐type, β1‐containing) versus *Kcnmb1* ^*−/−*^ (β1‐lacking) mice that were bred in‐house from the same parental pair. MCAs were *ex vivo* pressurized at 60 mmHg, the latter triggers development and maintenance of myogenic tone. In wild‐type male mouse MCA, fentanyl evoked dilation, as expected from BK channel activation, yet the concentration‐diameter response curve fails to follow a typical sigmoidal, with lower concentration of fentanyl (0.001–0.1 μm) leading to significant increase in MCA diameter and larger concentrations failing to do so (Fig. 2A,B). At these concentrations, fentanyl induced increase in diameter of ~3% from pre‐fentanyl levels. In contrast to males, cerebral arteries from females were resistant to low doses of fentanyl, yet a concentration‐dependent vasoconstriction is evident at higher fentanyl levels (0.1–30 μm) (Fig. 2C,D). MCA diameter response to increasing fentanyl concentrations could be fit with a sigmoidal function, rendering EC_50_ as 1, and EC_max_ as 10 μm fentanyl. At EC_max_, fentanyl‐driven constriction in MCA from females reached −4.2 ± 1.47% from pre‐fentanyl diameter.

**Fig. 2:**
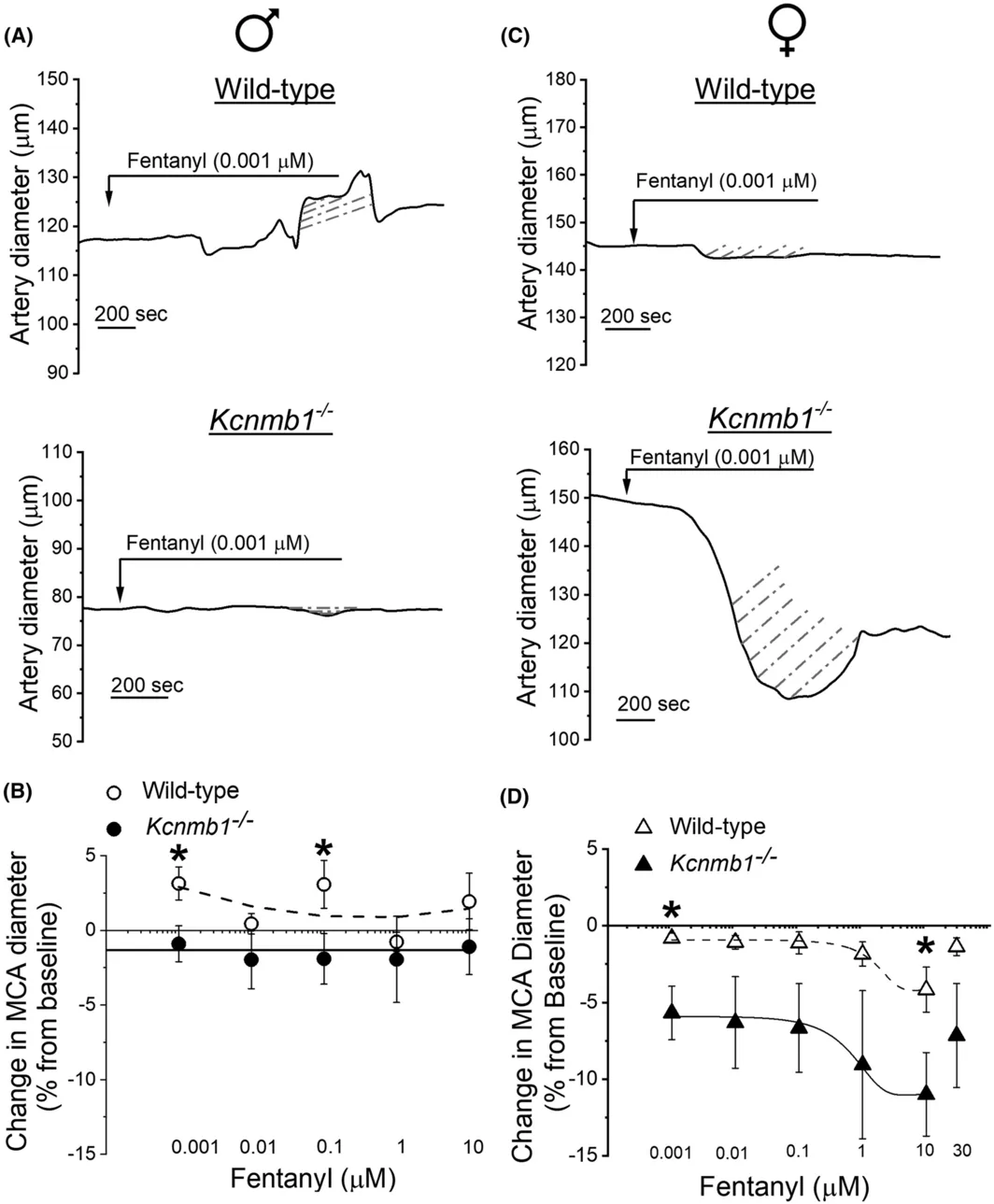
Calcium‐ and voltage‐gated potassium channel of large conductance (BK) β1 subunit participates in fentanyl effect on cerebral artery diameter. (A) Original trace showing changes in middle cerebral artery (MCA) diameter from male wild‐type (top) versus *Kcnmb1* ^*−/−*^ mouse (bottom). Here and in C, arteries were pressurized *ex vivo* using 60 mm Hg intraluminal pressure; dashed lines highlight fentanyl effect on diameter. (B) Averaged concentrations‐response plots from male MCAs showing percent‐change in artery diameter by fentanyl from pre‐fentanyl levels. Data presented as mean ± standard error of the mean (SEM). As the data did not conform to a sigmoidal Boltzmann function, a smooth curve was generated for visualization. *n* = 6–8 *P < 0.05 by two‐tailed Welch's *t*‐test. (C) Original trace showing changes in MCA diameter from female wild‐type (top) versus *Kcnmb1* ^*−/−*^ mouse (bottom). (D) Averaged concentrations‐response plots from female MCAs showing percent‐change in artery diameter by fentanyl from pre‐fentanyl levels. Data presented as mean ± SEM and fitted using sigmoidal Boltzmann function. *n* = 6–8 *P < 0.05 by two‐tailed Welch's *t*‐test.

Consistent with electrophysiological data underscoring the need for β1 functional expression for fentanyl to activate BK currents, in β1 subunit‐lacking (global knockout, *Kcnmb1* ^*−/−*^) male mouse MCAs, fentanyl vasodilation was lost (Fig. 2A,B). At 0.001 μm, fentanyl‐induced change in diameter averaged −0.88 ± 1.04% from pre‐fentanyl level. This is significantly different from fentanyl effect on diameter in wild‐type male mouse MCAs (*P* = 0.02 by two‐tailed Welch's *t*‐test). Remarkably, in female *Kcnmb1* ^*−/−*^ mouse MCAs, fentanyl application caused a concentration‐dependent, robust constriction (Fig. 2C,D). At EC_max_, that is 10 μm fentanyl in wild‐type females, fentanyl constricted *Kcnmb1* ^*−/−*^ female MCA up to −10.9 ± 2.71% from pre‐fentanyl diameter. This constriction is significantly higher when compared to fentanyl effect on diameter in wild‐type female mouse MCAs (*P* = 0.006 by two‐tailed Welch's *t*‐test). At lowest fentanyl concentration, 0.001 μm, arteries also constricted. This constriction averaged −5.68 ± 1.74% from pre‐fentanyl diameter, which was significantly higher than constriction by 0.001 μm fentanyl in MCAs from wild‐type females (−0.81 ± 0.32% from pre‐fentanyl diameter, *P* = 0.03 by two‐tailed Welch's *t*‐test). Collectively, data in this section indicate that BK β1 subunit participates in sexually dimorphic effect of fentanyl on MCA diameter.

### Critical role of BK β1 in fentanyl effect on cerebral artery diameter is mediated by both direct BK channel targeting and opioid receptor‐mediated pathways

BK β1 subunits may participate in fentanyl action on MCA diameter by two mechanisms that are not mutually exclusive. First, β1 may serve as a downstream target of the signaling cascade triggered by fentanyl‐driven activation of opioid receptors leading to modification of potassium conductance [42, 43]. Second, β1 may sense fentanyl in the absence of opioid receptor targeting (Fig. 1). To investigate these two possibilities, we first aim at establishing whether opioid receptor‐coding genes are expressed within mouse MCAs. We detect the presence of transcripts for all three types of opioid receptors: mu‐, kappa‐, and delta‐types in both males and females (Fig. 3). Next, we probed fentanyl effect on mouse MCA diameter in the presence of 10 μm naloxone. At these concentrations, naloxone blocks all types of opioid receptors [44, 45]. If fentanyl effect on MCA diameter is primarily triggered by opioid receptors, then fentanyl would lose the ability to dilate or constrict. Moreover, loss of BK β1 subunit would not modify the lack of fentanyl effect on diameter.

**Fig. 3:**
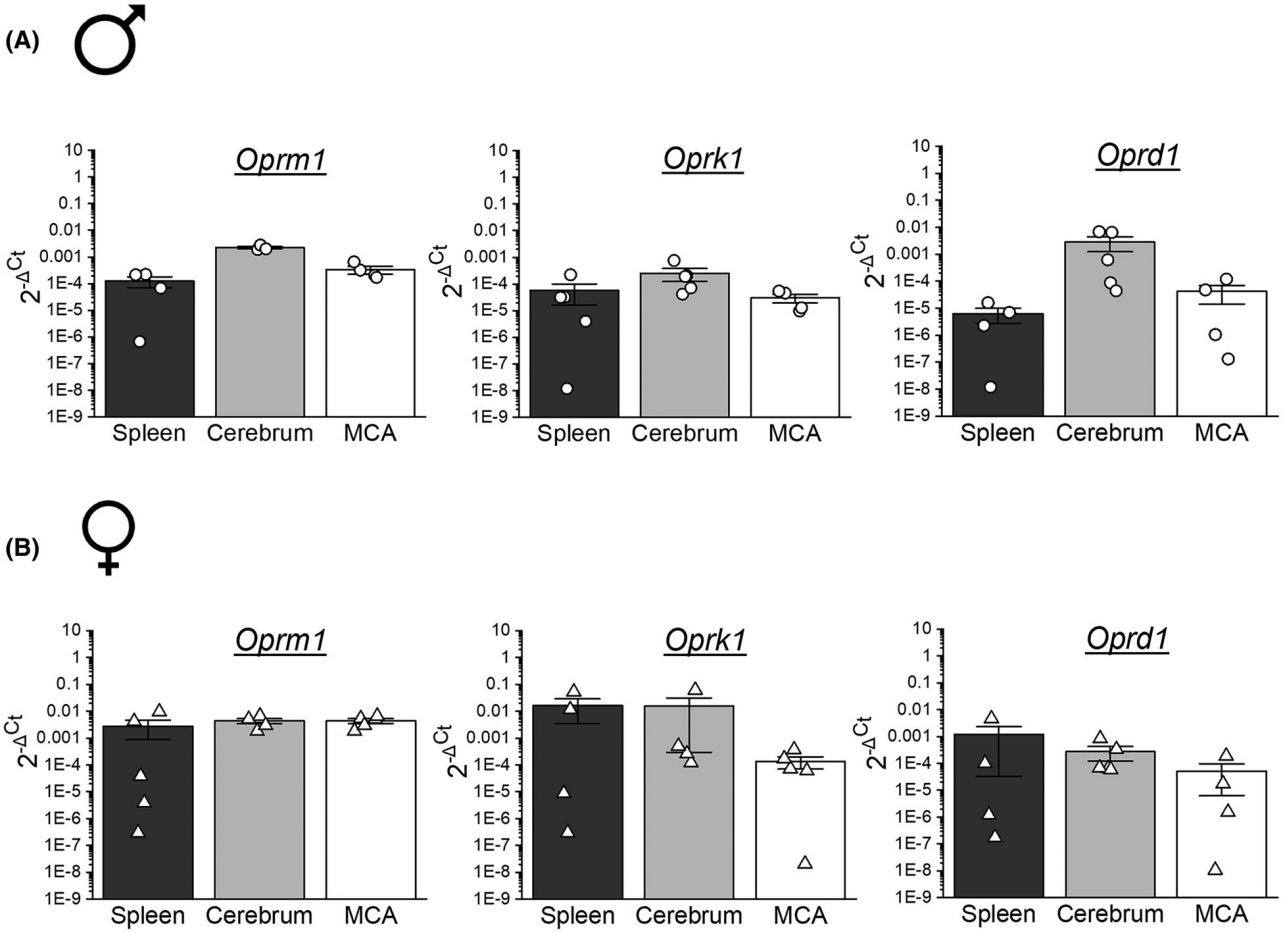
Opioid receptors mu, kappa, and delta (*Oprm1, Oprk1*, and *Oprd1*) transcript presence in C57BL/6J mouse tissue. Spleen and cerebrum were used as positive controls. TaqMan probes used included: Mm01188089_m1 (opioid receptor mu, *Oprm1*), Mm01230885_m1 (opioid receptor kappa, *Oprk1*), and Mm01180757_m1 (opioid receptor delta, *Oprd1*). Glyceraldehyde‐3‐phosphate dehydrogenase gene transcript *Gapdh* (probe Mm99999915_g1) was used as a reference gene. Gene expression levels were quantified using the 2^−Δ*C*t^ method [36, 37]. Error bars indicate standard error of the mean (SEM). (A) Data from males. (B) Data from females.

Indeed, pre‐treatment of MCAs from male and female mice with 10 μm naloxone ablates the effect of 0.001 μm fentanyl on diameter (Fig. 4). In males, fentanyl‐driven change in diameter in the presence of naloxone only averages 0.56 ± 0.17% from pre‐fentanyl levels (Fig. 4A,B). In females, it only averages 0.13 ± 0.34% from pre‐fentanyl levels (Fig. 4C,D). Two‐way ANOVA renders *P* = 0.017 for naloxone treatment as an independent variable in males, and *P* = 0.002 in females. However, in MCAs from *Kcnmb1* ^*−/−*^ males, 0.001 μm fentanyl causes mild constriction, which averages −1.1 ± 0.62% even in the presence of naloxone (Fig. 4A,B). The difference in fentanyl effect on MCA diameter in the presence of naloxone in male wild‐type arteries versus *Kcnmb1* ^*−/−*^ is statistically significant with *P* = 0.01086 (one‐tailed *t*‐test, Group 1 [wild‐type] > Group 2 [*Kcnmb1* ^*−/−*^]). In MCAs from *Kcnmb1* ^*−/−*^ females, 0.001 μm fentanyl causes constriction, which averages −0.78 ± 0.29% in the presence of naloxone (Fig. 4C,D). The difference in fentanyl effect on MCA diameter in the presence of naloxone in female wild‐type arteries versus *Kcnmb1* ^*−/−*^ is statistically significant with *P* = 0.03313 (one‐tailed *t*‐test, Group 1 [wild‐type] > Group 2 [*Kcnmb1* ^*−/−*^]). Thus, loss of β1 subunit still modifies fentanyl effect on MCA diameter even in the absence of opioid receptor signaling. Two‐way ANOVA renders statistical significance for the effect of genotype (loss of β1 subunit) as an independent variable: *P* = 0.0005 in males, and *P* = 0.003 in females. To establish whether the effect of β1 subunit loss is specific to a genetic manipulation or attributed to the loss of BK channel contribution to fentanyl effect in general, fentanyl was probed on MCAs from wild‐type (C57BL/6J) mice in the presence of the BK channel pharmacological blocker 1 μm paxilline [46, 47, 48]. In all cases, changes in artery diameter by 0.001 μm fentanyl were not statistically different from data obtained from *Kcnmb1* ^*−/−*^ mouse MCAs (Fig. 4B,D). In synthesis, we document the contribution of BK channel β1 subunit into modulation of cerebral artery diameter by fentanyl in the presence of opioid receptor blockade, thus likely involving direct fentanyl‐β1 subunit interactions.

**Fig. 4:**
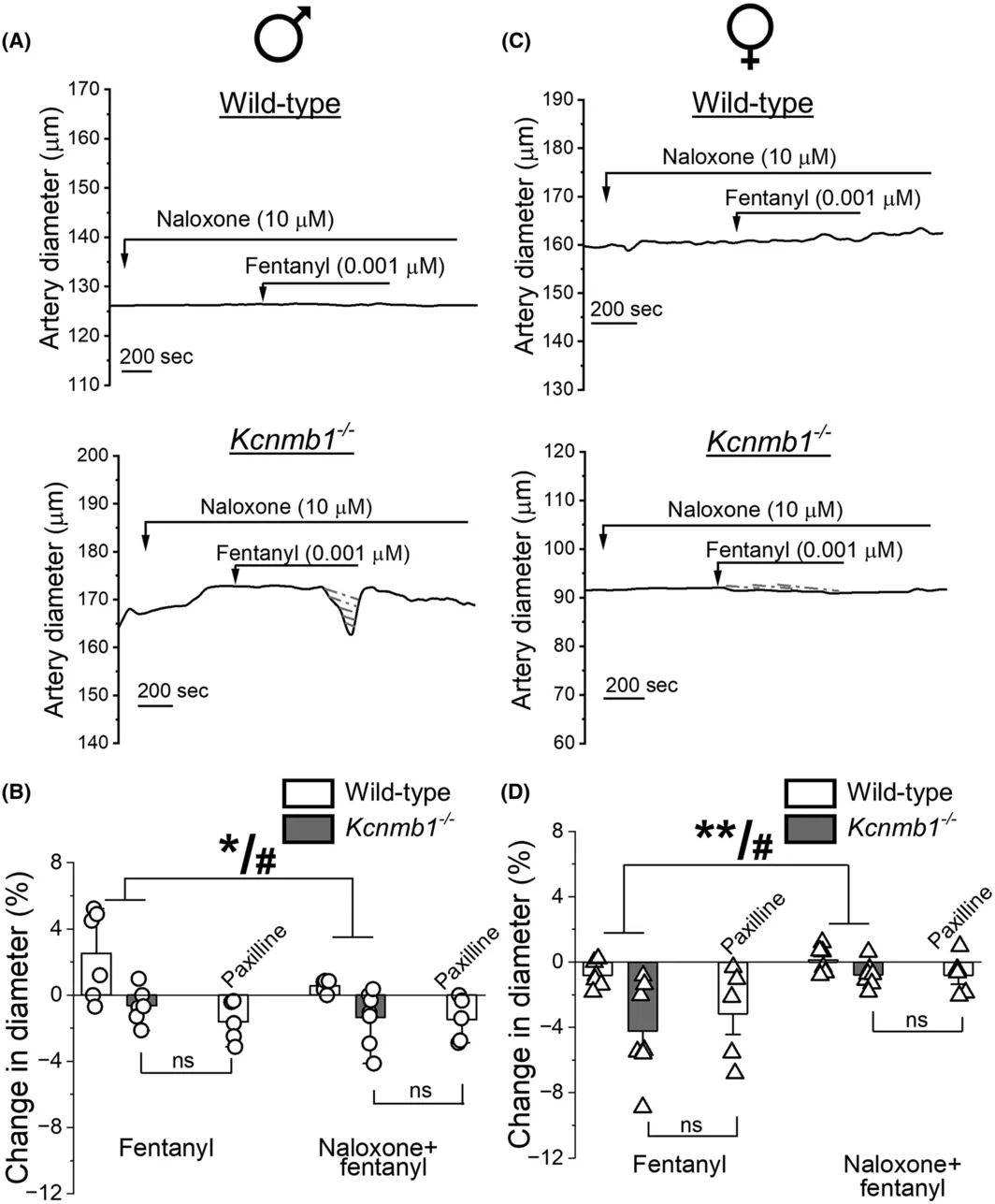
Loss of calcium‐ and voltage‐gated potassium channel of large conductance (BK) β1 subunit unveils fentanyl‐induced constriction in presence of opioid receptors blocker. (A) Original trace showing changes in middle cerebral artery (MCA) diameter from male wild‐type (top) versus *Kcnmb1* ^*−/−*^ mouse (bottom). Here and in C, arteries were pressurized *ex vivo* using 60 mm Hg intraluminal pressure; dashed lines highlight fentanyl effect on diameter. (B) Scattered graphs showing percent‐change in artery diameter from pre‐fentanyl level in male MCAs. Data is shown as mean + standard error of the mean (SEM). *n* = 5–7. *P < 0.05 by two‐way analysis of variance (ANOVA) for the effect of treatment (10 μm naloxone); #*P* < 0.01 by two‐way ANOVA for the effect of genotype (loss of BK β1 subunit in global genetic knockout *Kcnmb1* ^*−/−*^). Here and in D “ns” refers to the lack of statistically significant differences between changes in artery diameter by fentanyl in arteries from *Kcnmb1* ^*−/−*^ and wild‐type mice in presence of the BK channel blocker paxilline (1 μm). (C) Original trace showing changes in MCA diameter from female wild‐type (top) versus *Kcnmb1* ^*−/−*^ mouse (bottom). (D) Scattered graphs showing percent‐change in artery diameter from pre‐fentanyl level in female MCAs. *n* = 6–7. **P < 0.01 by two‐way ANOVA for the effect of treatment (10 μm naloxone); #*P* < 0.01 by two‐way ANOVA for the effect of genotype (loss of BK β1 subunit in global genetic knockout *Kcnmb1* ^*−/−*^). The effect of naloxone on the fentanyl‐induced MCA response did not differ significantly between male and female mice (*P* = 0.329, two‐tail Mann–Whitney *U*‐test).

## Discussion

In the last decade, several endogenous and exogenous compounds have been reported to modulate BK channel activity via β1 subunits, including estradiol, progesterone, lithocholic acid, leukotriene B4, ethyl alcohol, sodium 3‐hydroxyolean‐12‐en‐30‐oate (HENA), and celastrol [27, 28, 29, 30, 31, 49]. None of them, however, are opioids. While FDA‐approved anti‐diarrheal agonist of mu‐opioid receptor loperamide was reported to inhibit BK channels consisting of slo1 subunits and their combination with accessory gamma1 protein [21], our present work for the first time documents that the BK β1 subunit serves as an opioid sensor. Out of previously discovered β1 subunit‐dependent BK channel modulators, estradiol, progesterone, lithocholic acid, leukotriene B4, celastrol, and HENA are activators of BK channels [27, 28, 29, 30, 31, 49] while alcohol is a β1 subunit‐dependent inhibitor [28]. They do not act via a universal site on BK β1. On one hand, lithocholic acid, leukotriene B4, celastrol, and HENA are recognized by Thr169, while estradiol and progesterone partially overlap in their sensing amino acids, both requiring Trp163 [31, 34]. Whether fentanyl is recognized by Trp163 or Thr169 remains to be determined. Moreover, whether fentanyl acts on BK β1 as opposed to other members of the β family of BK accessory subunits (β2–4) remains to be established. In addition to direct sensing of fentanyl, β1 subunit may enable an increase in BK current by enabling sensing of fentanyl action on a third‐party component within BK channel surrounding.

Notably, the concentration‐response curve for increase in macroscopic BK current amplitude was best fitted with the biHill (biphasic Hill equation) function (Fig. 1D). This implies the existence of two overlapping processes that drive fentanyl's effect on BK currents, such as direct binding of fentanyl to β1 and opioid sensing of a third‐party mechanism, existence of two binding sites on β1, etc. Intriguingly, progesterone was recently shown to activate BK channel via sequential binding to β1 subunit's high‐ and low‐affinity sites [31]. Whether similar logic applies to fentanyl action should be addressed using binding studies. EC_50_ of fentanyl in our CHO cell culture experiments is 0.015 μm, which is lower than the EC_50_ (≈0.1 μm) reported to trigger Gi protein activation in a cell line following mu‐opioid receptor expression [50]. Thus, experimental evidence favors the idea that BK channels have high sensitivity to fentanyl, consistent with the notion that the β1 subunit possesses a high‐affinity binding site(s) for this opioid. In CHO cells, however, the activatory action of fentanyl on homomeric BK channels was observed at higher concentrations (≥ 0.1 μm) than those in native cells (i.e., MCA smooth muscle cells from *Kcnmb1* ^*−/−*^ mouse). Whether BK channel‐forming subunits possess low‐affinity sensing site(s) for fentanyl remains to be explored.

β1 subunit increases the apparent calcium‐sensitivity of BK channel, allowing BK to exert its negative feedback on smooth muscle contraction in cerebral arteries, uterus, bladder, and airways [23]. The physiological role of β1 in vascular smooth muscle is underscored by the fact that arterial constriction from BK block is blunted in β1‐lacking mice, which show both increased basal smooth muscle tone [25] and increased vascular fibrosis in response to high‐fat diet [51]. Expression pattern of β1‐coding gene enables BK channels to control physiology in a tissue‐selective manner and becomes an appealing therapeutic target to control cerebral artery diameter without major modulation of BK in non‐smooth muscle tissues [23, 52]. In our study, fentanyl effect on cerebral artery diameter from wild‐type (C57BL/6J) mice was minimal (Fig. 2). Indeed, fentanyl concentrations below lethal level were only able to dilate MCAs from males (Fig. 2B), this dilation did not exceed 5%. However, by Poiseuille's law, volumetric flow rate is proportional to the radius of the pipe (vessel) raised to the fourth power. Thus, a 5% increase in diameter is expected to increase brain irrigation by 22%. Notably, the fentanyl concentration showing a significant effect on *ex vivo* MCA does not correspond to concentrations where fentanyl activated BK currents in the recombinant cbv1 + β1 channel system. This difference may reflect the greater complexity of intact cerebrovascular tissue, as well as differences in BK channel subunit stoichiometry and/or regulatory environment between heterologous overexpression and native expression. In native vascular smooth muscle, BK channel activity is influenced by intraluminal pressure, local Ca^2+^ signaling, G protein‐coupled receptor‐dependent signaling, and interactions with other vascular cell types. Indeed, a study showed that BK channel activity measured in isolated cells may not fully predict BK‐dependent vasodilation in intact cerebral arteries when the complexity of the whole organ is present [53].

Loss of BK β1 subunit in both males and females resulted in vasoconstriction by fentanyl. BK channels are important regulators of myogenic tone [24], and *Kcnmb1* ^*−/−*^ mice are characterized by increased myogenic tone of cerebral arteries [25]. Increased myogenic tone in *Kcnmb1* ^*−/*−^ is expected to decrease constrictive responses to drugs as the artery is already constricted in response to intraluminal pressure more than the wild‐type counterparts. In our studies, however, we observed the largest constriction to fentanyl in *Kcnmb1* ^*−/−*^ arteries (Fig. 2B,D). This argues against the possibility that β1 controls response to fentanyl via modulation of basal myogenic tone.

Vasoconstriction by fentanyl was apparently larger in MCAs from *Kcnmb1* ^*−/*^ *−* females when compared to males (Fig. 2B,D). Fentanyl‐induced constriction in arteries from *Kcnmb1* ^*−/−*^ female mice, exceeding 5% (Fig. 2D), is expected to dampen brain perfusion profoundly. The reasons for sexual dimorphism of fentanyl action remain elusive. First, it could be attributed to sexual dimorphism of BK β1 abundance in arteries from males versus females. Yet apparently different sensitivity of MCAs from *Kcnmb1* ^*−/−*^ mice to constriction by fentanyl (Fig. 2B versus Fig. 2D) argues against such a possibility. Second, the reason could involve well‐known modulation of BK channel expression and function by female sex hormone estradiol [54]. Third, opioid receptors that mediate fentanyl effect may exhibit a sexually dimorphic profile of activity, as sex differences in opioid receptor‐mediated effects have been reported [55].

While BK channel function in cerebrovasculature has been reported to exhibit sexual dimorphism [56], apparent differences in fentanyl effect on MCA diameter between males and females disappeared in the presence of naloxone (Fig. 4B versus Fig. 4D). This argues in favor of opioid receptor‐mediated sexual dimorphism in fentanyl action. Linking sexual dimorphism to a specific receptor type is beyond the current scope of our work. We detected all three opioid receptor‐coding genes being expressed within mouse MCA (Fig. 3). Their functional contribution in control of artery diameter, however, may not be straightforward and remains to be established [57]. Notably, the block of opioid receptors with naloxone does not add to or synergize with the fentanyl‐induced vasoconstriction observed in the presence of the BK channel blocker paxilline or in *Kcnmb1* ^*−/−*^ mouse MCAs (Fig. 4B,D). This result indicates that opioid receptor signaling and BK channels may share a common pathway determining fentanyl action. BK β1 subunit emerges as a likely player in this common pathway.

Our findings clearly establish that fentanyl targeting of BK β1 subunit may modulate cerebral artery diameter independent of naloxone‐sensitive opioid receptors. Difference in EC_50_ for fentanyl activation of β1 subunit‐containing BK currents (0.015 μm, Fig. 1D) and constriction of β1‐containing MCAs from female wild‐type mice (1 μm, Fig. 2D) favors the idea that at the organ level, fentanyl‐sensitive BK currents are not the sole contributors to regulation of vessel diameter. Albeit the difference in EC_50_ between cultured CHO cells and the whole organ may also arise from differences in BK channel subunit stoichiometry (overexpression versus native expression profile). Interestingly, however, fentanyl did not inhibit β1‐lacking channels only consisting of cbv1 homomers expressed in CHO cells (Fig. 1C) while β1‐lacking MCAs were constricted by fentanyl (Fig. 2B,D). This outcome suggests that in the artery, loss of β1 unmasks fentanyl‐driven constriction, which is mediated by naloxone‐insensitive pathway(s). This constriction could potentially be explained by fentanyl‐driven inhibition of β1‐lacking BK channels. This explanation is plausible as mu‐opioid receptor agonist loperamide was reported to inhibit homomeric BK channels stably expressed in human kidney embryonic cells [21]. However, evaluation of fentanyl effect on artery diameter in presence of BK channel blocker paxilline rendered exactly the same results as *Kcnmb1* ^*−/−*^ (Fig. 4B,D). This means that the residual effect on artery diameter observed in presence of naloxone and paxilline does not involve BK channels and requires further investigation.

The differential outcomes in opioid action between stably expressing CHO and human embryonic kidney (HEK) cell cultures set a precedent that channel cellular environment may tune pharmacological responses. Indeed, differences in secretory pathways have been reported between CHO and HEK cells [58]. Additional explanations may include likely different pre‐ and post‐translational modifications between two cell lines; these modifications are known to modify BK channel function and pharmacology [59].

From a physiological standpoint, we identified BK β1 subunit as a novel protein sensor for opioids in the cerebrovasculature. One of the potential confounders of our study was the use of isoflurane for mouse anesthesia. Isoflurane is an anesthetic that is considered to have high solubility in aqueous media (blood) [60]. As such, its washout time from the tissue is highly dependent on the duration of exposure [60]. After 15 min‐long exposure, less than 15% of isoflurane remains in the blood by 20 min post‐exposure, while only 10% of isoflurane remains in the blood by the 40‐min mark [60]. In our hands, however, isoflurane exposure only was 30 s‐long and was followed by 20 min of equilibration for temperature and minimal intravascular pressure (10 mmHg) before myogenic tone development was triggered. It took another 35 min to obtain stable myogenic tone at 60 mmHg across sexes and genotypes tested. Considering the extremely brief exposures to isoflurane during our experiments and reported rapid washout times of this anesthetic, we do not expect that large quantities of isoflurane remained in cerebral artery tissue when fentanyl was probed.

Another potential confounder is the use of genetic β1 knockout (*Kcnmb1* ^*−/−*^). Lifelong absence of *Kcnmb1* may trigger compensatory adaptations that by themselves may be responsible for alterations in fentanyl action observed in arteries from *Kcnmb1* ^*−/−*^. While such compensatory mechanisms cannot be excluded, the similarity of fentanyl effect on artery diameter in *Kcnmb1* ^*−/−*^ arteries with fentanyl effect in presence of acute BK channel block with paxilline (Fig. 4B,D) argues against non‐specific action of genetic knockout on our experimental outcomes.

Since BK β1 subunits have been reported to have a differential expression profile across major cerebral arteries [61], our findings may contribute to the variant effects of fentanyl on cerebral circulation in different brain regions. Moreover, our findings warrant follow‐up studies to probe the role of the β1 subunit in opioid action on smooth muscle in organs besides cerebral arteries, such as the uterus, bladder, and trachea. Lastly, the protective action of BK β1 against fentanyl‐induced constriction of cerebral arteries makes this subunit an attractive therapeutic target against fentanyl‐associated ischemia.

## Conflict of interest

The authors declare no conflict of interest.

## Author contributions

AB conceived and designed the project, AG, ST, and MN acquired data, AG, ST, MN, and AD analyzed and interpreted the data, AB wrote the paper. All authors edited the manuscript and participated in weekly discussions leading to manuscript completion.

## Data Availability

Original recordings and datasets are available upon request to the corresponding author.
